# TRPV6 as A Target for Cancer Therapy

**DOI:** 10.7150/jca.31640

**Published:** 2020-01-01

**Authors:** John M. Stewart

**Affiliations:** Soricimed Biopharma Inc. 18 Botsford Street, Moncton, NB, Canada, E1C 4W7

**Keywords:** TRPV6, calcium, cancer, prostate, breast, pancreas.

## Abstract

Two decades ago a class of ion channels, hitherto unsuspected, was discovered. In mammals these Transient Receptor Potential channels (TRPs) have not only expanded in number (to 26 functional channels) but also expanded the view of our interface with the physical and chemical environment. Some are heat and cold sensors while others monitor endogenous and/or exogenous chemical signals. Some TRP channels monitor osmotic potential, and others measure cell movement, stretching, and fluid flow. Many TRP channels are major players in nociception and integration of pain signals. One member of the vanilloid sub-family of channels is TRPV6. This channel is highly selective for divalent cations, particularly calcium, and plays a part in general whole-body calcium homeostasis, capturing calcium in the gut from the diet. TRPV6 can be greatly elevated in a number of cancers deriving from epithelia and considerable study has been made of its role in the cancer phenotype where calcium control is dysfunctional. This review compiles and updates recent published work on TRPV6 as a promising drug target in a number of cancers including those afflicting breast, ovarian, prostate and pancreatic tissues.

## Background

### Transient Receptor Potential Channels

It is rare that a discovery opens an unexpected and unsuspected window on how animals, including humans, interact with their physical and chemical environments. A revolution in understanding the interface between animals and environment (external, extra-tissue, extracellular) began with the discovery in *Drosophila melanogaster* 1, 2 of what are now called the transient receptor potential channels. This discovery was rapidly followed by reports of a transient receptor potential channel in mammals 3, 4 that resembled the vanilloid receptor (VR1) reported earlier 5. Since then this mammalian ion channel superfamily has expanded to include 26 functional members in six subfamilies: TRPC (canonical), TRPA (ankyrin), TRPML (mucolipin), TRPM (melastatin), TRPP (polycystin) and TRPV (vanilloid) 6-9.

Gradual appreciation of TRP channels can be traced through review articles that act as milestones in understanding their physiological function 6, 9-19, regulation by associated proteins 20-22, evolution 23, intracellular trafficking 24, 25, pre-mRNA splicing 26, and interactions with immune cells 27. Monographs of the TRP field have also appeared 28-31. The last specific review of the role of TRPV6 in cancer was published in 2012 32 with reference to cancer in reviews of larger scope 23, 33, 34. This review summarizes recent work on TRPV6 as it emerges as a therapeutic target in solid cancers derived from epithelia.

### Transient Receptor Potential Vanilloid channels

The vanilloid sub-family of TRP (TRPV) channels comprises six members, TRPV1 to TRPV6. The first four channels are related in sequence (average homology about 45% 35) and are activated variously by heat, acid, stretching/osmotic strain, and certain exogenous chemicals (e.g. capsaicin), and also play roles in nociception and pain signal integration 8, 9, and thermoregulation 36. Although related by sequence homology to the first four channels (~35%)^35^, TRPV5 and TRPV6 have greater sequence homology to each other (81%) 35, and show significantly different channel properties than the 'sensory' channels. TRPV5 and TRPV6 are more selective for calcium ion (P_Ca_/P_Na_ ~ 100) compared to the other four TRPVs (P_Ca_/P_Na_ ~1 to ~15), show minimal, if any, voltage or heat dependence, and are constitutively active 8, 37. The major function of these TRPV5 and TRPV6 at the whole organism level appears to be coarse calcium homeostasis. In mice TRPV5 is expressed predominantly in the kidney where it reclaims calcium from the pre-urine stream 38 at the level of the distal tubules. TRPV6 is predominant in the gastro-intestinal tract where it has a role in calcium import, initiating the process by importing calcium ion through the apical membrane 3, 39, 40. The homeostatic role of the two calcium-sensitive channels in human renal intestinal and tissues has been reviewed 41.

### TRPV6 protein in normal human tissues

TRPV6 protein has been reported variously in placenta, salivary gland, prostate, pancreas, testes, liver and lung but not without great inconsistency. This is not surprising considering that a reliable antibody to TRPV6 is not commercially available. Healthy human prostate produces very little, if any, TRPV6 42, 43. Human lung tissue appears to produce low levels of TRPV6 protein as shown in isolated lung epithelial cells 44, 45. TRPV6 protein is produced in human placenta 46, 47, and the protein shows up-regulation in human endometrium during the menstrual cycle 48 and the later stages of pregnancy when fetal bone mineralization occurs 46. Human duodenum expresses TRPV6 mRNA but TRPV6 protein was not examined 49, 50. TRPV6 protein was not detected in human liver 40. Salivary gland shows TRPV6 mRNA 43 and the protein was detected in the basolateral membrane of acinar cells 51. Many of these works corroborate the earlier immunohistochemical detection of TRPV6 in esophageal epithelia, and the small and large intestine, the exocrine pancreas with weak staining in the acinar cells 40. This latter work also indicated there was TRPV6 staining of ductal epithelia of breast and sweat gland and that in all cases staining was considerably weaker than in malignancies of the examined tissues.

Supplementary Table S1 shows a compendium of RNA-Seq data from four studies 52, 53, 54, 55 that report the amount of TRPV6 mRNA (in Transcript Per Million, TPM) that encompassed 296 subjects and, in total, 91 putatively healthy tissues. Excerpted from this Supplementary Table S1 are those tissues and studies that showed TPM values greater than 0.5, the cut-off point for these experiments for (minimally) triplicate determinations (Table 1). These data can be found at the Expression Atlas Database (www.ebi.ac.uk) (https://www.ebi.ac.uk/gxa/experiments/) where expression is considered as low (0.5 to 10 TPM), medium (11 to 1000 TPM) and high (>1000 TPM). The median expression values, with few exceptions, are wildly different across the four studies. The largest value is 56 TMP (for prostate) but this tissue is also reported as 0 TPM and 17 TPM. Nevertheless, it is clear that the expression of TRPV6 mRNA is low is most tissues. The most consistent result was for pancreas, even though the amounts are not large (10 TPM, 0 TPM, 24 TPM and 26 TPM). For comparison, in esophagus mRNA levels of TRPV6 and two common calcium-binding proteins (S100A11, Calmodulin 1) show 2 TPM, 2997 TPM and 343 TPM as median values respectively 52 and β-actin shows 3724 TPM when TRPV6 is 3 TPM 55.

Immunohistological data for TRPV6 protein in tissues are not as available as mRNA data and, as expected, more difficult to quantify and report. The Human Protein Atlas (www.proteinatlas.org) reports protein expression for assorted human tissues. For TRPV6 staining a polyclonal rabbit antibody (registry number AB-2684885) was used. This antibody is cited as 'Approved' out of a ranking sequence of Enhanced, Supported, Approved and Uncertain so results must be interpreted in this light, particularly given the database cites this antibody as “partly consistent with gene/protein characterization data”. Table 2 has been excerpted from the Human Protein Atlas database to provide a comparison to tissue-specific TRPV6 mRNA in cancers illustrated in Figure 2. To appreciate the difficulty in scoring the IHC staining Supplementary Figure S1 illustrates Not Detected, Low, Medium and High ranking for some tissues. The only tissue to produce a ranking of High TRPV6 was placenta that is very dynamic and dependent on the stage of pregnancy 46.

Studies with TRPV6 knock-out mice highlighted the involvement of this channel in calcium homeostasis 56 producing defective intestinal Ca^+2^ absorption, reduced fertility, and increased urinary calcium. A key role for TRPV6 in male fertility and maturation of sperm in murine models with non-functional or excised TRPV6 has been reported 57, 58 and show decreased calcium, mediated by TRPV6, is required for sperm maturation in the lumen of the epididymis.

### Structure of the TRPV6 channel

**Figure 1:**
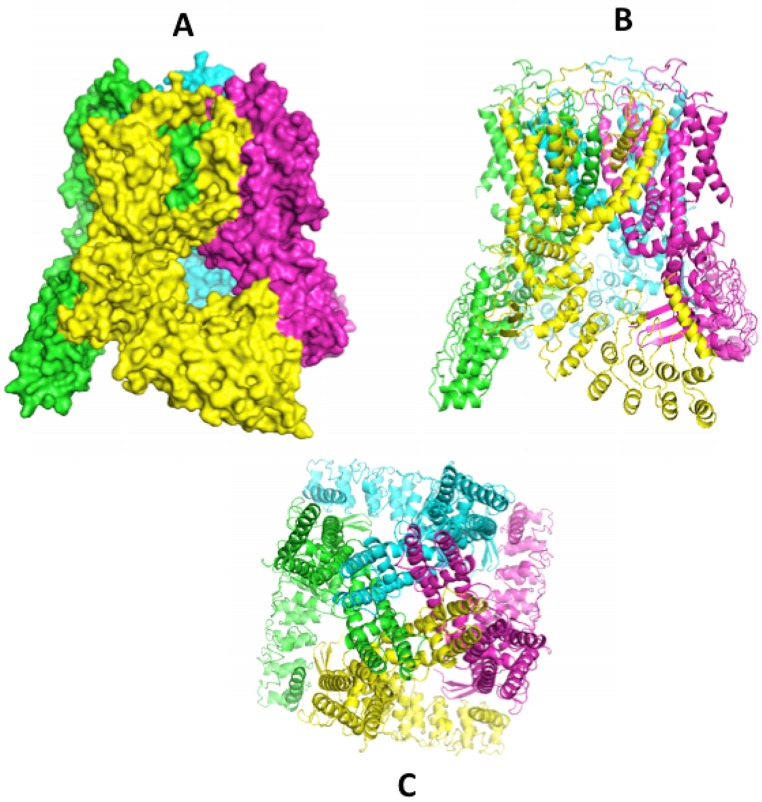
Structures of hTRPV6 determined by cryo-EM in nano-disks (ref 61) 61. Image A (space filled) and B (ribbon) show a side view with monomer units in different colours clearly showing the domain swapping of helices S5-S6 of a 'preceding' monomer seen in green against the yellow focus monomer and the yellow S5-S6 interacting with the 'following' purple monomer. The bottom image (C) shows the top view, looking down the axis of the ion pore. Structures can be accessed at the RCSB Protein Data Base (www.rcsb.org) code 6BO9 61.

A flood of data published recently gives a clearer picture of the complex 3-D structures of the TRPV family of channels. Recent papers from Sobolevski's group at Columbia University have outlined the architectural elegance of the homotetrameric structures, along with information on the ion pore structure. First was an X-ray crystal structure for modified rat TRPV6 in 2016 59. Following quickly were rat 60 and human TRPV6 structures 61 by cryo-EM. The earlier rat X-ray structure was corrected after domain swapping between monomer units was uncovered 62. Further studies of the pore structure followed 61, 63 and further emphasized the importance of the calcium gating function of the aspartate (D541) residue reported earlier 64. These major accomplishments have now been extended to the first structure for the only remaining unsolved TRPV channel, TRPV3 65. These structures join the previous reports for TRPV1 66, TRPV2 67, 68, TRPV4 69 and TRPV5 70. The structure of slightly modified human TRPV6 (code 6BO9) 61 is shown in the series of images in Figure 1 accessed through the RCSB Protein Data Bank (www.rcsb.org). With insight from this collection of molecular structures we can reasonably expect advances in molecules designed to modulate the activities of TRPV ion channels. For example, the promiscuous TRPV modulator 2-APB (2- aminoethoxydiphenyl borate) has been modeled to a binding site in TRPV6 for which it is an inhibitor 60 and to a TRPV3 site where it is an agonist 65. Econazole, an inhibitor of a number of ion channels 71 including TRP channels has been modeled to a binding site on this TRPV5 70. One common point of these identified binding sites is that they access the 'top' face of the channel and as such would not likely effect channels in the gut that face the lumen where calcium ions (and other divalent ions) are captured.

### Calcium and cancer

Calcium plays a central role in development and maintenance of cancer phenotypes. Elevated cellular calcium as well as microcrystals of calcium salts in cancer cells are linked to malignancies and metastasis in breast cancers 72, 73. Temporal, spatial and/or amplitudinal alterations of internal calcium concentrations influence gene transcription, tumourigenesis, cell proliferation, metastasis and susceptibility to apoptosis 74-76. Calcium-dependent remodeling of the tumour microenvironment influences angiogenesis, tumour progression and recruitment of macrophages 77. The centrality of calcium in cancer can be seen in the multiple roles of the calcium-binding protein sorcin and its role in angiogenesis, migration, apoptosis, multidrug resistance and invasion 78. Recent reviews of targeting calcium signaling in cancer therapy emphasize TRPV6 activity 79, 80. All these linkages of calcium to various aspects of the cancer phenotype implicate TRPV6.

### TRPV6 as an oncochannel

The over-expression of TRPV6 mRNA and protein has been reported in a number of human malignancies 14, 81. TRPV6 has been classified as an oncochannel 82 and its gene as an oncogene 32, 83, 84 although there is no evidence expression of TRPV6 itself can induce cancer or proto-oncogenes 85. An early report showed high TRPV6 mRNA in a colorectal cancer cell line (SW480), a human chronic myelogenous leukemia cell line (K-562) 86, 87 and rat leukemia cells 88. Up-regulation of TRPV6 mRNA was shown in prostate cancer 43 and in prostate cancer cell lines LNCaP and PC3 89. It should be noted that expression of TRPV6 in PC3 and DU145 cells *in vitro* appears to be inconsistent. In prostate tumours, a positive correlation between the Gleason score and TRPV6 mRNA has been reported 87. Immunohistochemistry of TRPV6 in healthy and malignant tissues showed low (if any) levels of protein in healthy exocrine tissues (e.g. mammary gland, pancreas, prostate) but elevated amounts in breast, colon, ovary, prostate and thyroid carcinomas 40. Correlations of TRPV6 over-expression and Gleason scores extended to extra-prostatic extensions 42 and a role for TRPV6 in predicting prostate malignancies was suggested as TRPV6-positive tumours often invade extra-prostate tissues 90, 91 with a poor prognosis. The exact role of TRPV6 in cancer proliferation is not clear, but calcium-dependent proliferation of cancer cells was linked directly to TRPV6 92.

Breast cancer also shows increased TRPV6 mRNA and has been reported to be 2 - 15-fold greater in breast cancer when compared to healthy tissue 93, 94. TRPV6 protein was elevated more in invasive tumour areas over non-invasive tumour areas in 93.3% of biopsies 94. Elevated TRPV6 was reported in estrogen receptor-negative breast cancers, and correlated to poor prognosis 95. Reducing TRPV6 production with siRNA in breast (T-47D) 93, and prostate cancer cell lines (LNCaP) 96 resulted in decreased cell proliferation and increased apoptosis.

This calcium channel plays a role in gastrointestinal cancers particularly at early stages. TRPV6 mRNA is at very low levels (if at all) in late stage tumours (Stage III and IV) while 66% of Stage I tumours, and 17% of Stage II tumours show the channel over expressed 97. There is a report that capsaicin treatment leads to TRPV6-dependent apoptosis in a gastric adenocarcinoma cell line (AGS) because of increased intracellular calcium 98. Perhaps this result is because of a gross increase in calcium (instead of increased calcium transients) that could lead to calcium toxicity and activation of the apoptotic circuit. This effect may be confounded by the discovery of viral infection of AGS (ATCC CRL-1739) by parainfluenza type 5 (PIV5) as reported by the ATCC after its discovery 99 in AGS where the virus resulted in increased degradation of STAT1.

**Figure 2:**
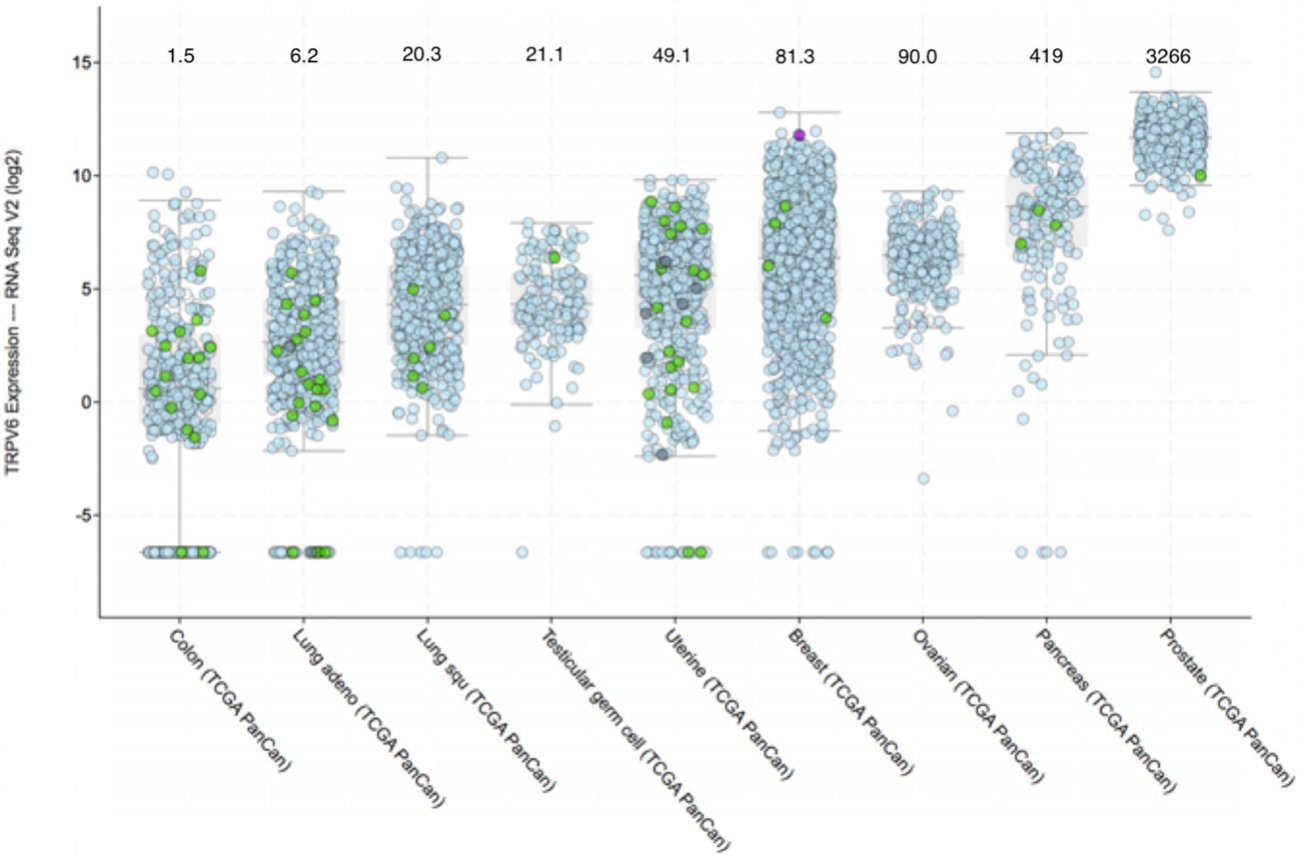
Prevalence of TRPV6 mRNA expression in various tumours according to the TCGA Pan-Cancer database. Cancer types are sorted by median value of fold change over healthy tissue. RNA-SEQ expression data were extracted from the cBioPortal (www.cbioportal.org) 165, 166. Quantitation of TRPV6 mRNA expression was done using the RNA-SEQ next generation sequencing. Results are in log2 scale meaning that +1 is up regulated (2^1^ = two-fold) compared to normal samples of each study. Values that are less than 0 indicate down regulation. The colour and form of each dot represent the mutational status of TRPV6 gene as given in the figure. Median values for fold change above normal tissue can be estimated from the interactive graphic constructed by the database and are as follows and are shown above the cancer type: The full cancer descriptions, in order of appearance from left to right are: Colon adenocarcinoma; Lung adenocarcinoma; Lung squamous carcinoma; Testicular germ cell carcinoma; Uterine Corpus Endometrial Carcinoma; Breast invasive adenocarcinoma; Ovarian serous cystadenocarcinoma; Pancreatic adenocarcinoma; Prostate adenocarcinoma.

Figure 2 shows the expression of TRPV6 mRNA in various solid cancers. Ovarian, prostate and pancreatic cancers are particularly noted because a large proportion of the tumors (>90%) consistently express TRPV6 mRNA well above normal levels.

Ovarian cancer was cited in an early study as over-producing TRPV6 protein in one biopsy 40, but only recently was a survey of the five different types of ovarian cancer (low grade serous, high grade serous, clear cell, endometrioid, mucinous) reported 100. As always, care should be taken in assessing reported amounts of TRPV6 protein since there is often difficulty with available antibodies. Elevated TRPV6 mRNA was reported in early and late stages of all five of the disease types classified under the umbrella of ovarian cancer when compared to healthy tissue. Immunohistochemical detection of TRPV6 protein in tissue microarrays for ovarian cancers likewise showed elevated protein in all cancer types and at early and late stages of the diseases with little (if any) in biopsies of healthy tissue. Targeting TRPV6 with TRPV6-specific, antagonistic peptides reduced growth of SKOV-3 tumour xenografts in mice 100 further supporting this channel as a viable target. TRPV6-binding peptides delivered a conjugated fluorescent label to TRPV6-rich xenografts of human ovarian cancer (SKOV-3) and prostate cancer (DU 145), and superparamagnetic nanoparticles to SKOV-3 tumours suggesting the diagnostic photo-imaging and MRI-imaging potential of this over-expressed channel 101.

TRPV6 was recently implicated directly in development and prognosis of pancreatic cancer with decreased survival in patients with elevated tumour TRPV6 protein levels 102. Reducing TRPV6 protein production in pancreatic cell lines with siRNA reduced proliferation and invasion, and initiated apoptosis and cell cycle arrest 102. In a Phase I clinical trial of a peptide inhibitor of TRPV6 activity two patients with advanced pancreatic cancers showed tumour reduction (one by -27% by RECIST criteria) with one patient showing 55% decrease in the validated pancreatic cancer biomarker CA19-9 103. Of the 23 patients enrolled in this study with cancers classed as “TRPV6-rich” >50% showed stable disease after two courses of treatment with no drug-related serious adverse events.

TRPV6 has been reported down regulated in some cancers. Cervical squamous cell carcinoma, in early stages, showed decreased levels of TRPV6 mRNA and protein 104. In 145 resected non-small cell lung cancer patients, decreased TRPV6 protein levels were reported as associated with shorter median survival times 105. Esophageal squamous cell carcinoma patients showed down regulated TRPV6 mRNA and protein but with no correlation between these changes and disease specific survival (DSS) although for a short, 3-year DSS there was a small negative effect with male patients and a positive effect with female patients 106. It is too early to know if such decreases in TRPV6 are a result of or involved in the development and progression of squamous cell neoplasms.

### Mechanism of action of TRPV6 as an oncochannel

Elevated TRPV6 and subsequent sustained increases in cytosolic calcium activates the nuclear factor of activated T-cells (NFAT) transcription factors in cell lines of prostate 96 and breast cancers 93. In these studies reduction of TRPV6 expression with silencing RNA reduced proliferation and increased apoptosis. Over-expression of TRPV6, which is constitutively active, results in a sustained elevation of intracellular calcium, which is required for activation of the calmodulin/calcineurin/NFAT pathway. The short half-life of dephosphorylated NFAT in the nucleus (~15 - 20 min) 107, 108 requires consistent elevated cytosolic calcium to create what has been interpreted as a survival response against cell death, or a mechanism to decode calcium oscillation into a build-up of dephosphorylated NFAT in the cytoplasm 109. While the response in each cancer type would be specific to that cancer, cell line etc. because of a different cohort of accessory transcription factors and other proteins that could interact with NFAT, the literature provides a general outline for a mechanism of action.

A target of Ca^+2^/calmodulin-activated calcineurin (a phosphatase) is NFAT, a hyper-phosphorylated transcription factor that is activated by dephosphorylation 110. The role of NFAT in regulating the cell cycle and apoptosis was reviewed recently 111 as has its role in cancer 112. Activated NFAT translocates to the nucleus 113 where it modulates a number of genes in partnership with Jun/Fos and other proteins. Activated genes influence proliferation and migration and include Membrane Type 1 Matrix Metalloproteinase and Matrix Metalloproteinase-type 2 114, and autotaxin 115, 116. Autotaxin is a secreted phospholipase that produces lysophosphatidyl choline, a ligand of a Growth Factor Receptor and lysophosphatidic acid receptor 1 117. The reported anti-apoptotic nature of increased TRPV6 may results from increased production of Bcl-2, an anti-apoptotic protein that inhibits the release of cytochrome c from mitochondria and prevents apoptosome formation 118. As well, hydroxyapatite microcrystals common in such nodularized tumours, presumably because of increased calcium influx, up regulate MMPs 73. This simplistic signalling pathway description would be much more complicated because of four NFAT isoforms (excluding a calcineurin-independent one), a plethora of genes that are regulated and modulated by NFAT 111 and about 30 other proteins with which NFAT can partner in transcription complexes 111. The roles of NFATs in cancer development and as a potential therapeutic target have been reviewed 112, 119-122.

A detailed study of the involvement of TRPV6 in a number of prostate cancer cell lines showed increased trafficking of TRPV6 to the plasma membrane, dependent on the Orai1 protein of the Store-Operated Calcium Channel 85. Accompanying increased TRPV6 trafficking was increased cell proliferation, reduced apoptosis and greater cisplatin resistance 85. With this clearer focus on the molecular details, these authors suggest TRPV6 activity is a survival response of at least three prostate cancer cell lines (LNCaP, PC-3 and DU 145) implicating a sensitivity to and requirement of increased intracellular calcium transients compared to normal tissue in the cancer phenotype. While the literature provides hints of the role of TRPV6-related elevation of intracellular calcium in the oncology phenotype and, conversely, what its inhibition might do, downstream effects of TRPV6 inhibition on cancer cell signalling pathways and gene expression are just beginning to emerge. Using siRNA to knock down TRPV6 expression in the capan-2 cell line model of pancreatic cancer resulted in significant decrease in Bcl-2 (B-cell lymphoma 2, apoptosis inhibitor) and increase in BAX (Bcl-2-associated X protein, a promoter of apoptosis) which, taken together, indicate activation apoptosis 123. Additionally, significant decreased MMP9 expression (matrix metalloproteinase 9) suggests decreased metastasis, and decreased PCNA expression (Proliferating cell nuclear antigen) suggests decreased DNA synthesis; the observed significant increase in calcium-dependent E-cadherin expression may play a role in cell-cell interaction.

### Control of TRPV6 expression

It is now well established that a number of epithelial type cancers over-express TRPV6 mRNA and likely overproduce protein compared to healthy tissues. How the TRPV6 gene becomes over expressed is not known. There is much work to do in this area but some influences are summarized below.

### Involvement of Vitamin D and p38α^MAPK-14^ in TRPV6 expression

Vitamin D (D3) activates the production of TRPV6 ion channel. The VDR (Vitamin D receptor, a nuclear receptor) bearing Vitamin D binds to response elements on the TRPV6 gene, activating transcription 124. VDR is an obligate heterodimer with retinoic acid receptor alpha in its active form 125. More than 1000 Vitamin D Response Elements have been noted across the human genome 126, 127 and 3000 across the murine genome 127 providing for very complicated signalling and cross signalling. But, the role of Vitamin D is complicated further since TRPV6 also has a reciprocal role in how Vitamin D3 influences cancer 128.

Recently, a closer view of this has emerged revealing another factor involved in the Vitamin D-influenced increase TRPV6 transcription. Vitamin D also activates the transcription of GADD45α (Growth arrest and DNA damage-inducible protein alpha) that is also produced in response to either DNA damage or stressed growth arrest conditions 126. GADD45α activates MEKK4 (Mitogen Activated Protein kinase kinase kinase) that in turn activates p38α (aka CSBP2 or MAPK-14α) and JNK (c-Jun N-terminal kinase). While p38α, a 38 kD protein kinase activated by phosphorylation, is produced in response to cell stress, it also enhances Vitamin D-dependent TRPV6 transcription beyond Vitamin D alone 126. Inflammatory cytokines (e.g. IL-6) can activate p38α that, by phosphorylating histone-3, contributes to the chromatin relaxation status 129, potentially allowing for greater access to TRPV6 gene. On the other hand, p38α has been referred to as a tumour suppressor 130 although it has been connected to proliferation in a number of cancer cell lines such as breast cancer 131, 132, chondrosarcoma 133, prostate neoplasms 134, melanoma 135 and a number of others including HeLa cells 136.

Activated JNK inhibits NFAT4 (NFATc3) and NFATc2 (NFAT1) thus allowing for greater apoptotic activity 126, presumably by phosphorylating c-Jun and reducing formation (or activity) of the NFAT/c-Jun/Fos complex on DNA. The latter is unclear since phosphorylated c-Jun has been reported as more active in some studies 137. For cancer cells, it is possible enhanced TRPV6 production could counteract reduced NFAT activity. A recent review of p38α pathways has been published 129 summarizing these interactions.

Factors in addition to Vitamin D can also be expected to influence TRPV6 expression. In Vitamin D Receptor null, pregnant mice, TRPV6 was depressed, but in pregnant VDR-null mice duodenal TRPV6 mRNA increased about 13-fold 138.

While Vitamin D and TRPV6 have been linked to various pathologies arising from calcium deployment, the most familiar effect is that of bone metabolism and in particular osteoporosis 13. It is interesting then that TRPV6 inhibitors can also reduce bone resorption in models of osteoporosis 139.

### TRPV6 transcription is regulated by other nuclear receptors

The control of TRPV6 production by androgen receptor (AR) was first reported in LNCaP cells 96. While providing evidence that downstream signalling from elevated calcium concentration is through the NFAT system, these authors showed that knocking down AR with siRNA resulted in decreases in TRPV6 mRNA by 48 h and TRPV6 protein by 72 h post treatment. The authors suggest AR is a co-regulator of TRPV6 transcription rather than having a direct role. The role of AR in TRPV6 was cited again in terms of antagonist and agonist of the receptor 32 where dihydrotestosterone, an AR agonist, inhibits TRPV6 expression while an AR antagonist (bicalutamide) increases TRPV6 expression 89, 140, 141. As well, androgen treatment of LNCaP decreases TRPV6 mRNA by 80% in one day 89. In androgen sensitive prostate cell line LNCaP, TRPV6 expression has also been reported to be sensitive to AR, but in a ligand-independent manner 96. While the presence of an androgen response element in the 5' flanking region of the TRPV6 gene was suggested 142 there are no supporting data, but there may be a 'tier two' Androgen Receptor response element that is intergenic and at position -13,232 of the TRPV6 gene 143.

Estrogen receptor (ER) has been linked to TRPV6 expression through a response element in the gene 144. Tamoxifen, an estrogen receptor antagonist, resulted in down regulation of TRPV6 mRNA in breast cancer cell line T-47D while estrogen increased it, as did progesterone and estradiol 93. Estrogen-dependent up regulation of TRPV6 in breast and prostate cancer has also been reviewed and suggested to act in concert with other transcription factors that are activated by elevated calcium 142. During rat pregnancy both uterine and placental levels of TRPV6 are up regulated by progesterone receptor and estrogen receptor dependent pathways, and is decreased by antagonists to these receptors 145.

Another nuclear receptor, Peroxisomal Proliferator Activated Receptor alpha (PPARα), has a response element on the TRPV6 gene 146 although any effects of its ligands (polyunsaturated fatty acids, endocannabinoids, fibrates) on TRPV6 status are not known. There may be a link between PPARα, endocannabinoids such as anandamide and TRPV channels, including TRPV6, during oxidative stress 147. Since PPARα, like Vitamin D, also requires dimer formation with retinoic acid receptor α, it provides for an interesting question of the links of retinoids and fatty acids to cancer.

### TRPV6 expression regulated by transcription factors

There are a large number of binding sites for transcription factors (TF) in the promoter region of TRPV6 gene 148. The list of curated transcription factors cited by this database contains HOXA5 MAZ, NKX2-1, PPARA, TLX2, and ZEB1. Uncurated associations of TFs regulating *trpv6* expression from this site, and using known binding site sequences comprise GATA1, GLI2, HNF1A, KLF13, MTF1, NFE2, NR5A2, RBPJ, and VDR. None of the transcription factors listed has been reported in the literature as affecting *trpv6* expression with the exception of VDR and PPARα, while AR and ER, missing from the database entry, have been (see above). All of the transcriptions factors listed above have been implicated in some aspect of the oncologic process.

### Location: TRPV6 expression in tumours varies with cell location

Expression of TRPV6 mRNA and protein depend on the local environment of the cell and, in tumours, cell position. In a study of 140 prostate tumours TRPV6 was strongly expressed in those tumour cells in contact with the stroma, and strong band-like patterns were seen were the cells were in contact with the normal tissue 42. This locational production of TRPV6 was also reported in a study of other prostate tumours 90 and to a degree in prostate cancer cells 85. In breast cancers, tumour staining for TRPV6 protein revealed a more general staining throughout the lesion but with more intense staining on the margins in contact with normal tissue 94. In this latter study there was greater TRPV6 expression (3 - 4X) in smaller (≤2 cm) than in larger (>2 cm) Stage I breast tumours, but about the same in both size categories of Stage III tumours and showed particular concentration of the channel in invasive regions. As a word of caution for work with cancer cell lines, the level of expression of TRPV6 may increase with both time in culture and passage number as observed in equine chondrocytes where TRPV6 increased 4-fold over 3 passages 149.

### Gene copy number

Over expression of TRPV6 may result from amplification of the number of TRPV6 genes. Normally there should be a copy number of 2, but TRPV6 gene amplification has been reported in breast cancer cell lines and biopsies 95. An examination of genetic data on the www.cbioportal.org database for gene amplification in each of the cancer types listed in Figure 2 showed amplification of the TRPV6 gene was not a common occurrence. The rate of gene amplification was less than 1% of cancers in Figure 2 except for ovarian serous cystadenocarcinoma (1.7%) and prostate adenocarcinoma (1.0%). Overall the incidence of TRPV6 gene amplification was 33 gene duplications in 4,517 tumours (0.7%) and thus, is not likely a common cause of TRPV6 expression changes.

The cbioportal database (www.cbioportal.org) can be used to determine whether TRPV6 gene copy number correlates with increased TRPV6 mRNA. For breast adenocarcinoma (n = 1020, r = -0.06) and pancreatic adenocarcinoma (n = 184, r = -0.09) there are no statistically significant Pearson's correlations. On the other hand, ovarian cystadenocarcinoma (n = 585, r = 0.29, p < 0.001) and prostate adenocarcinoma (n = 494, r = 0.36, p < 0.001) showed statistically significant correlation between TRPV6 mRNA and TRPV6 gene copy number. Thus, the case is mixed whether amplification leads to greater TRPV6 mRNA transcription.

### Gain/Loss-of-function mutations leading to pathology

There has been one putative gain-of-function TRPV6 mutation reported and connected to pathology 150. This mutation, consisting of three, non-synonymous polymorphisms (C157R; M378V; and M681T) had significantly greater representation in renal calcium stone patients than in non-stone forming patients. This single report of pathology in an ancestral homozygotic is surprising considering the low levels (if any) of TRPV6 protein in human kidney. This mutation, initially reported in the seminal publication of human TRPV6 gene 43 was also reported as an ancestral type 151. The TRPV6 sequence from one patient homozygous for the ancestral haplotype was expressed in *Xenopus* eggs and showed double the calcium permeability compared to the derived haplotype 150. On the other hand the channel expressed in HEK293 showed no statistical difference between calcium conductance and other parameters 152. There are a number of SNPs reported 43, 150 but none have been linked to increased TRPV6 function or a disease state. In fact, the two expressed proteins TRPV6a (R157, V378, T681) and TRPV6b (C157, M378, M681) showed no difference in the frequency of expression in prostate cancer patients 153 indicating TRPV6 alleles have no differential influence on prostate cancer.

Work is beginning to emerge on pathologies from loss-of-function mutations of TRPV6. A maternally inherited, heterozygous, missense mutant (G660R) and a paternally inherited, non-sense mutant (R510Ter) resulted in severe interference in mineralization of the fetal skeleton 154. Transient neonatal hyperparathyroidism with associated difficulties in maternal-fetal calcium transport has been reported for loss-of-function mutations including a frame-shift mutation, three missense mutations and a combined frame-shift plus intronic mutation that altered mRNA splicing 155.

### Additional Factors

Mature TRPV6 is heavily N-glycosylated 156 but exists in a few major forms: a fully glycosylated ion channel and an ion channel with modified or absent oligosaccharide. TRPV5 and TRPV6 expressed in HEK293 cells are activated by fluid flow and removal of the glycan from TRPV5 resulted in loss of this phenomenon 157. Klotho, a β-glucuronidase linked to ageing, is believed to play a part in initiating the deglycosylation of TRPV5 and TRPV6 158. Removal of sialic acid residues capping the oligosaccharide attached to TRPV5 by Klotho increases its residence time in the membrane 159 through interaction with galectin 159. Treatment with a Klotho also causes increased calcium flux in TRPV6 160, 161. Silencing Klotho produced no change in TRPV6 expression in bone marrow-derived dendritic cells 162. The role of Klotho in cancer is not clear since it has been reported as down regulated in breast cancer 163, up regulated in ovarian cancer 158, and both up and down regulated in various hepatocellular cancers 163. The glycosylation status of TRPV6 tetramers may play a role in the overall 3D structure of those TRPV and may also influence what channel modulators can do to the channels and to where on the channel they bind. Constitutively active, TRPV6 may offer an opportunity to study the effect of glycation on large polymeric protein complexes, carrying as it does its own built-in monitor (calcium influx).

### Pharmacology of TRPV6

TRPV6 has emerged as a target in cancer treatment because of its role in increasing intracellular calcium and initiating downstream signalling pathways that increase cell proliferation, metastasis and inhibition of apoptosis. As such, TRPV6 joins the list of ion channels that are being targeted for cancer treatment. A review of pharmacological approaches to exploiting TRP channel activity in cancer has been published 164 and a number of TRPV6 inhibitors are cited in a recent review of targeting calcium signalling in cancer 79. A peptide inhibitor of TRPV6 (SOR-C13) completed a Phase I clinical safety trial 103 and has been shown to reduce growth in cell and animal models ovarian and prostate cancers 100, 101. Table 3 summarizes the public literature for inhibitors of TRPV6 and TRPV5.

## Summary

TRPV6 is clearly a valid target to disrupt further the aberrant calcium homeostasis observed in and required by many cancers. Reduction of TRPV6 activity by decreasing expression of the channel or by pharmacological intervention has shown efficacy in four cancer types: adenocarcinomas of breast, ovarian, prostate and pancreas. The evidence for the utility of TRPV6 inhibition in solid cancers has built over the last number of years in cancer cell lines, in xenograft murine models, and now has a suggestion of efficacy in humans. Whether targeting TRPV6 with antibodies or antibody drug conjugates, peptide inhibitors or peptide drug conjugates, or new chemical entities informed by 3-D structural analysis, there is a path forward to exploit the over production of TRPV6 to our benefit. The key issues to be resolved for a successful antagonist of TRPV6 are: an IC_50_ low enough to be clinically meaningful (i.e., required dose), specificity in inhibiting only TRPV6, low human toxicity, bio-stability, and cost. Still, it is unclear how the expression of TRPV6 gene and production of protein is up regulated in these cancers and that may offer another route to exploit TRPV6.

## Supplementary Material

Supplementary figures and tables.

## Figures and Tables

**Table 1:** RNA-Seq data for TRPV6 mRNA in normal human tissues excerpted from Supplementary Table 1.

	Experimental Accession Number			
	E-MTAB-2836 52	E-MTAB-3358 http://fantom.gsc.riken.jp/5/	E-MTAB- 4344 53	E-MTAB- 5214 55
Tissue	Median (N = 122)	Median (N = 96)	Median (N = 25)	Median (N = 53)
	TPM	TPM	TPM	TPM
Brodmann (1909) area 24				2
Brodmann (1909) area 9				3
C1 segment of cervical spinal cord				4
Breast		0		1
Caudate nucleus				2
Cerebral cortex	2	0		3
Endocervix				0.6
Esophagus mucosa				3
Esophagus	2	0		
Gall Bladder	23	0		
Hippocampus proper				0.6
Minor Salivary Gland		0		5
Nucleus Accumbens		0		1
Occipital Lobe		2		
Ovary	0.2	0.5	0	0
Pancreas	10	0	24	26
Penis		4		
Pituitary Gland		0		0.6
Placenta	17	0		
Prostate	56	0		17
Putamen				2
Saliva-secreting gland	37	0		
Small Intestine	0.2		16	
Stomach	2			3
Substantia Nigra			0	1
Suprapubic Skin		0		8
Testes	1		0.7	2
Thyroid Gland	3			1
Urinary Bladder	1	0		2
Vagina		0		2
Zone of Skin	13			

**Table 2:** Ranking IHC of normal human tissues for TRPV6 staining (Human Protein Atlas: www.proteinatlas.org). ND indicates Not Done.

Tissue	Cell Type	TRPV6 IHC Ranking
Breast	Adipocyte	Low
	Glandular Cells	ND
	Myoepithelial cells	Low
Colon	Endothelial Cells	Medium
	Glandular Cells	ND
Duodenum		ND
Epididymis		Low
Liver		ND
Lung	Pneumocytes	Low
Ovary	Stroma Cells	Low
Pancreas		ND
Placenta	Decidual cells	High
	Trophoblastic cells	ND
Prostate		ND
Salivary Gland		ND
Seminal Vesicle		Medium
Small Intestine		ND
Spleen		ND
Testes	Leydig Cells	Medium
	Seminiferous Ducts	Medium

**Table 3:** Summary of compounds that inhibit TRPV5 and/or TRPV6. **Abbreviations:** LNCaP & PC-3-prostate cancer cell lines; VG = voltage gated; 2-APB = 2-aminoethyl-diphenylborate; IP3 = Inositol 1,4,5-triphosphate; SERCA = Sarcoplasmic/Endoplasmic Reticulum Ca^+2^-ATPase; Orai = protein component of Calcium Release Activated Channel (CRAC); SOCE = Store Operated Calcium Entrance channel; cyt P450 = cytochrome P450; MTD = Maximum Tolerated Dose.

Compound	TRPV6 IC_50_ (μM)	TRPV5 IC_50_ (μM)	Other targets & comments	Efficacy/ Toxicology	Clinical Development
Ruthenium Red	9 167, 168	0.12 168	N-type VG Calcium channels 169; TRPV2 170; in thapsigargin negative experiments, inhibition of Ca is only 60% 171; not bound to pore 167. Suppresses CRACs 172	Neurotoxic 169	None reported
TH-1177	675 173	456 173	IC_50_ = 3.2 μM in LNCaP & 17 μM in PC-3 174. T-type VG Calcium channels 175; TRPC1 176	None in mice at 180 mg/kg 174	None reported
TH-117 best derivative	90 173	503 173	Other TRP channels 173	No information,	None reported
2-APB	20.7 177	No data	CRAC channel IC50 = 10 uM 178. IP3 receptor, SERCA, Orai, various TRP channels 177. TRPV6 inhibition is allosteric 60	rapid hydrolysis & transesterification 177	None reported
2-APB derivative 22b	5 177	No data	SOCE, IC_50_ = 2.8 μM 177	Inhibits CRAC channels essential to T-cells at 10 mM 178	None reported
Econazole	201 173	442 173, 168	Cyt P450 179; VG Calcium channels, 179; receptor operated Calcium channels 71	Broad-spectrum antifungal agent.^180^	Commercial antifungal: Spectrazole (USA), Ecostatin (Canada)
Miconazole (monistat)	201 173	442 173	TRPV4 181; cyt P450 inhibitor 182	Antifungal agent^180^	Commercial antifungal: Monistat, Micatin
Piperazine derivative Cis-22a	0.32 183	2.4^183^	TRPV1, TRPV3, TRPV5, TRPM8, SOCE 183	*In vitro* growth inhibition of TRPV6-containing cell line T 47D^183^	None reported
Capsaicin	Estimated as between 25 uM and 50 uM for apoptosis from Figure 1 of 98	Not tested	Inhibits TRPV6 in human SCLC and increases apoptosis *in vitro* and decreases murine xenografts 45. Increases apoptosis in gastric cancer cells 98.	Well-recognized activator of TRPV1 heat sensor.	None reported
Δ^9^-tetrahydro- cannabivarin (THCV)	9.4 184	4.8 184	TRPV5, TRPV6 inhibition 184; Agonist to TRPV3 and TRPV4 185; Activated TRPV1, TRPA1, TRPV2 186.	None reported	None reported
SOR-C13 (13 amino acid peptide)	0.014 101	No effect	Reduces ovarian tumour xenografts 100.	No drug-related serious adverse clinical events, no MTD determined, safe to 6.2 mg/kg 103	Completed Phase Ia clinical trial 103
Lidocaine	Not done but from viability graphs ~5 mM 187	Not Done	Reduced cell invasion and migration of MDA-MB-231, PC-3 and ES-2 cells. 187	None reported	None reported.

## Abbreviations

TRPV6: Transient Receptor Potential Vanilloid 6
TRPV5: Transient Receptor Potential Vanilloid 5
VR1: vanilloid receptor 1
TRPA: Transient Receptor Potential Ankyrin
TRPC: Transient Receptor Potential Canonical
TRPM: Transient Receptor Potential Melastatin
TRPML: Transient Receptor Potential Mucolipin
TRPP: Transient Receptor Potential Polycystin
TPM: Transcripts Per Million
cryo-EM: Cryogenic electron microscopy
2-ABP: 2- Aminoethoxydiphenyl borate
RECIST: Response Evaluation Criteria in Solid Tumors
NSCLC: Non-small cell lung cancer
DSS: disease specific survival
NFAT: nuclear factor of activated T-cells
Bcl-2: B-cell lymphoma 2
MMPs: matrix metalloproteinases
VDR: Vitamin D Receptor
GADD45α: Growth arrest and DNA damage-inducible protein alpha
MEKK4: Mitogen Activated Protein kinase kinase kinase
JNK: c-Jun N-terminal kinase
IL-6: interleukin 6
AR: Androgen Receptor
PPARα Peroxisomal Proliferator Activated Receptor alpha: PPARα
ER: Estrogen Receptor
