# Supplementary material for: TRPV6 as A Target for Cancer Therapy
Source: J Cancer. 2020 Jan 1;11(2):374–87. doi: 10.7150/jca.31640 (PMC6930427; doi:10.7150/jca.31640)
Supplement: Supplementary file 1 — Supplementary figures and tables. [file jcav11p0374s1.pdf]

Supplemental Figure S1

Ranking: NOT DETECTED

Duodenum Liver Pancreas Spleen

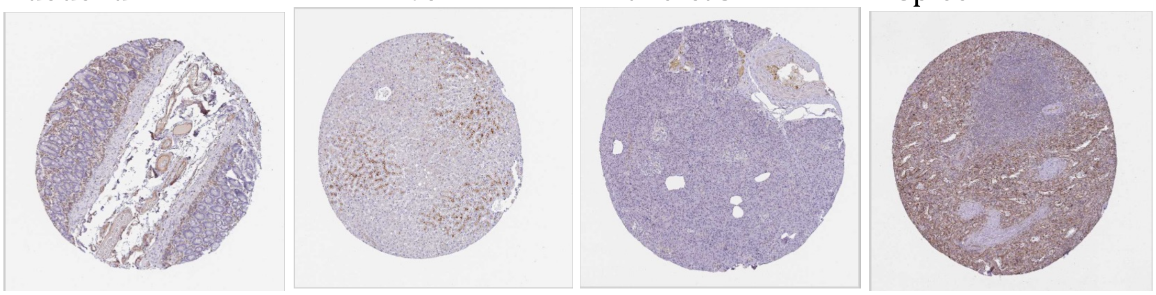

Ranking: LOW

Breast Epididymis Fallopian Tube Ovary

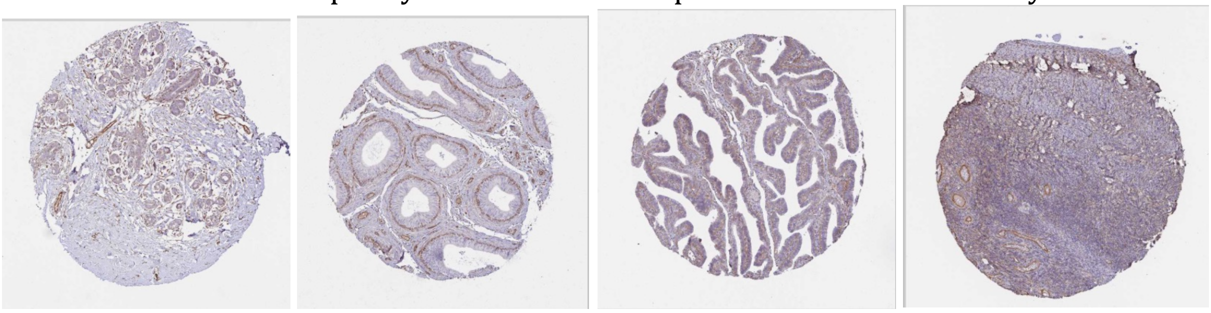

Ranking: MEDIUM

Endometrium Kidney Testes Thyroid

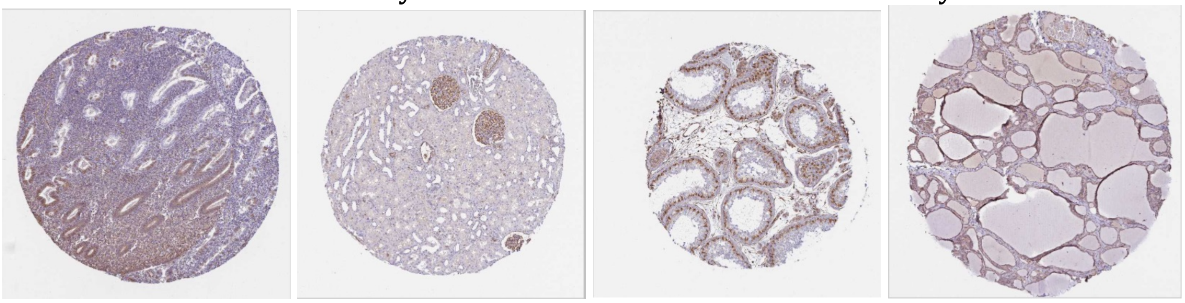

Ranking: HIGH

Placenta (decidual cells)

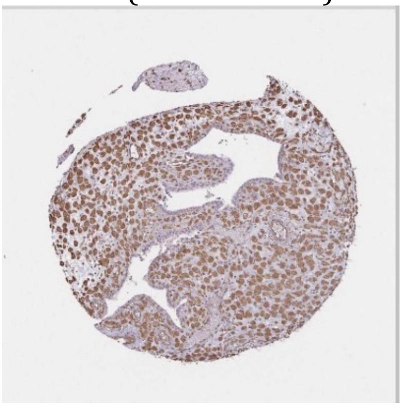

Experimental Accession Number	E-MTAB-2836	E-MTAB-3358	E-MTAB-4344	E-MTAB-5214
	n = 196	n = 96	n = 25	n = 53
Tissue	MEDIAN TPM	MEDIAN TPM	MEDIAN TPM	MEDIAN TPM
Brodmann (1909) area 24	N	N	N	2
Brodmann (1909) area 9	N	N	N	3
C1 segment of cervical spinal cord	N	N	N	4
EBV-transformed lymphocyte	N	N	N	0
adipose tissue	0.1	N	0	N
adrenal gland	0.1	0	0	0
amygdala	N	N	N	0
aorta	N	0	N	0
artery	N	0	N	0
bone marrow	0.1	N	N	N
brain	N	0	0	N
breast	N	0	N	1
caudate nucleus	N	N	N	2
cerebral cortex	2	0	N	3
cerebellum	N	0	N	0
colon	0.2	0	N	N
diencephalon	N	0	N	N
dorsal thalamus	N	0	N	N
dura mater	N	N	N	N
duodenum	13	N	N	N
ectocervix	N	N	N	0
endocervix	N	N	N	0.6
esophagogastric junction	N	N	N	0
esophagus mucosa	N	N	N	3
esophagus muscularis mucosa	N	N	N	0
esophagus	2	0	N	N
epididymis	N	0	N	N
fallopian tube	0.9	0	N	0.6
gall bladder	23	0	N	N
globus pallidus	N	0	N	N
greater omentum	N	0	N	0
heart	0	0	0	N
heart left ventricle	N	0	N	0
hippocampal formation	N	N	N	N
hippocampus proper	N	N	N	0.6
hypothalamus	N	0	N	0

left cardiac atrium	N	0	N	N
locus ceruleus	N	N	N	N
liver	0.1	0	0	0
lung	0.4	0	0	0
lymph node	0.2	0	N	N
medulla oblongata	N	0	N	N
middle frontal gyrus	N	0	N	N
middle temporal gyrus	N	N	N	N
minor salivary gland	N	0	N	5
mitral valve	N	0	N	N
nucleus accumbens	N	0	N	1
occipital cortex	N	0	N	N
occipital lobe	N	2	N	N
olfactory apparatus	N	0	N	N
ovary	0.2	0.5	0	0
pancreas	10	0	24	26
parietal lobe	N	0	N	N
parotid gland	N	0	N	N
penis	N	4	N	N
pineal body	N	0	N	N
pituitary gland	N	0	N	0.6
placenta	17	0	N	N
prostate gland	56	0	N	17
pulmonary valve	N	0	N	N
putamen	N	N	N	2
rectum	0.2	N	N	N
saliva-secreting gland	37	0	N	N
seminal vesicle	N	0	N	N
sigmoid colon	N	N	0	0
skeletal muscle tissue	0	N	N	0
small intestine	0.2	N	16	N
small intestine Peyer's patch	N	0	N	0
smooth muscle tissue	0.4	N	N	N
spinal cord	N	0	0	N
spleen	0.1	N	0	0
stomach	2	N	N	3
subcutaneous adipose tissue	N	0	N	0
submandibular gland	N	0	N	N
substantia nigra	N	N	0	1
suprapubic skin	N	0	N	8
testis	1	N	0.7	2

thyroid gland	3	N	N	1
tibial artery	N	N	N	0
tibial nerve	N	0	N	0
tongue	N	0	N	N
tonsil	0.3	N	N	N
transformed skin fibroblast	N	N	N	0
transverse colon	N	0	N	0
tricuspid valve	N	N	N	N
urinary bladder	1	0	N	0
uterus	N	0	N	0
vagina	N	0	N	2
vas deferens	N	0	N	N
vermiform appendix	0.1	0	N	N
zone of skin	13	N	N	N
